# Pregnancy outcomes of PCOS overweight/obese patients after controlled ovarian stimulation with the GnRH antagonist protocol and frozen embryo transfer

**DOI:** 10.1186/s12958-018-0352-z

**Published:** 2018-04-10

**Authors:** Rui Chen, Shiping Chen, Manting Liu, Hua He, Haiyan Xu, Hanyan Liu, Hongzi Du, Weihua Wang, Xuefeng Xia, Jianqiao Liu

**Affiliations:** 1 Key Laboratory for Reproduction and Genetics of Guangdong Higher Education Institutes, Key Laboratory for Major Obstetric Diseases of Guangdong Province, Third Affiliated Hospital of Guangzhou Medical University, Guangzhou, 510150 People’s Republic of China; 2 Department of Reproductive Medicine, Third Affiliated Hospital of Guangzhou Medical University, Guangzhou, 510150 People’s Republic of China; 3 Institute for Viral Hepatitis, Key Laboratory of Molecular Biology for Infectious Diseases, Ministry of Education, Chongqing Medical University, Chongqing, 400010 People’s Republic of China; 4 Houston Fertility Laboratory, Houston, TX 77063 USA

**Keywords:** Polycystic ovary syndrome, Overweight, Obesity, GnRH antagonist protocol, Frozen embryo transfer, Pregnancy outcomes

## Abstract

**Background:**

Overweight/obese women with polycystic ovary syndrome (PCOS) are at increased risk of subfertility and complications of pregnancy, compared with normal-weight women. To implement controlled ovarian hyperstimulation (COH), the improved efficacy of the gonadotrophin-releasing hormone antagonist (GnRH-ant) protocol has been demonstrated, as well as frozen embryo transfer (FET).

**Objective:**

This retrospective study evaluated the pregnancy outcomes after combined GnRH-ant protocol and FET in overweight/obese women with PCOS, with reference to that of normal-weight women with PCOS.

**Methods:**

Women with PCOS (*n* = 398) who underwent the GnRH-ant protocol for COH followed by FET, were stratified as normal-weight (BMI < 24 kg/m^2^) or overweight/obese (BMI ≥24 kg/m^2^). The outcomes of pregnancy were compared.

**Results:**

The overweight/obese patients had significantly lower rates of embryo implantation (47.7%), live birth (47.8%), and live births of twins (10.9%) compared with the normal-weight group (58.4%, 60.8%, and 30.0%, respectively; *P* = 0.006, 0.015, and 0.000), while the rate of late abortion was significantly higher (11.0% cf. 3.8%, *P* = 0.030). BMI was the only significant factor affecting the probability of live birth.

**Conclusion:**

The pregnancy outcomes of overweight/obese women with PCOS after COH via the GnRH-ant protocol and FET remained at a significant deficit compared with that of normal-weight women with PCOS.

## Background

Polycystic ovary syndrome (PCOS) is the most common endocrine disorder among women of reproductive age [1]. PCOS is often concomitant with obesity, oligo- or amenorrhea, hormonal abnormality, and infertility [2, 3]. Obesity is also associated with complications of reproduction, including maternal mortality; stillbirth; neonatal and infant death; infant large-for-gestational age; fetal malformations; maternal diabetes; pregnancy-induced hypertension; pre-eclampsia; and Caesarean section [4–6]. Thus, obese women with PCOS are at increased risk of infertility and complications of pregnancy.

Patients with PCOS and infertility may resort, as final treatments, to assisted reproductive techniques, including controlled ovarian hyperstimulation (COH) and embryo transfer. COH implemented by the gonadotrophin-releasing hormone antagonist (GnRH-ant) protocol requires less time and is more cost-effective than the standard GnRH agonist long protocol, and is also associated with a significantly lower incidence of ovarian hyperstimulation syndrome [7, 8]. Thus, the GnRH-ant protocol is recommended for infertile women with PCOS. Furthermore, while fresh embryo transfer is standard for in-vitro fertilization (IVF)/intracytoplasmic sperm injection (ICSI), it has been demonstrated that pregnancies and live births that arise from frozen-thawed embryos have better obstetric and perinatal outcomes [9]. A multicenter, randomized, controlled trial confirmed that women with PCOS who underwent fresh embryo transfer experienced a significantly lower rate of live births than did those who were given frozen embryo transfer (FET) [10]. Thus, it may be expected that assisted reproduction that includes a combination of COH via the GnRH-ant protocol and FET may help to ease the risk of reproduction complications in women with PCOS, including those who are overweight/obese.

The present retrospective analysis evaluated the pregnancy outcomes of overweight/obese women with PCOS after treatment by the GnRH-ant protocol for COH and FET, by comparison to the outcomes of normal-weight women with PCOS.

## Methods

### Study population

This retrospective analysis reviewed the results of IVF/ICSI-FET performed from January 2014 through December 2016 at the Reproductive Medicine Center of Third Affiliated Hospital of Guangzhou Medical University, China. The Ethics Committee of the hospital approved the study. Because this was a retrospective investigation, patients were not asked to participate in the analysis.

The inclusion criteria were: patients with PCOS who underwent their first IVF/ICSI cycles and then primary FET cycles; maternal age < 35 years; and use of the GnRH-ant protocol for COH. A total of 398 cycles (patients) were analyzed. For analysis, the pregnancy outcomes of patients with normal weight (N-W; *n* = 260) were compared with that of overweight/obese patients (O-O; *n* = 138), where the threshold was a body mass index (BMI) of 24 kg/m^2^ [11, 12].

The definition of PCOS was in accordance with the Rotterdam criteria [13], therefore fulfilling ≥2 of the following: oligo- or anovulation; clinical or biochemical signs of hyperandrogenism; and polycystic ovaries and exclusion of other etiologies (i.e., congenital adrenal hyperplasia, androgen-secreting tumors, or Cushing’s syndrome).

### Study procedures

All 398 patients received the GnRH antagonist protocol for COH, oocyte retrieval, and fertilization, followed by cryopreservation, freeze-thaw, and transfer of embryos.

Ovarian stimulation with recombinant human follicle-stimulating hormone or human menopausal gonadotrophin was initiated on day 2 or 3 of the menstrual cycle. Doses were adjusted according to the ovarian response, as monitored via ultrasonography and measurement of serum sex steroids. For the fixed protocol, GnRH-ant (Cetrorelix, Mercerono) at a daily dose of 0.25 mg was initiated in the morning of day 5 or 6 of stimulation, and continued up to the day before triggering ovulation, to inhibit a premature surge in luteinizing hormone surge. For the flexible protocol, GnRH-ant at a daily dose of 0.25 mg was initiated, when the largest follicle exceeded 12-14 mm. Human chorionic gonadotropin (hCG) at a dose of 4000-10,000 IU was administered to induce oocyte maturation, when ≥2 follicles measured ≥18 mm. Oocyte retrieval was performed 34-36 h later.

Oocytes were inseminated approximately 4-6 h after follicular aspiration by IVF or ICSI, depending on sperm quality. Morphologic criteria were used for embryo scoring. After 3 to 6 days of culture, high-quality embryos were frozen by vitrification.

Endometrial preparation for every FET cycle was performed according to the natural cycle, artificial cycle, or induced ovulation cycle. For natural cycles, follicular growth was monitored by serum hormones and ultrasound beginning on cycle day 10. Endometrial thickness > 7 mm was chosen for embryo transfer. For patients with irregular menstrual cycles after ovarian stimulation, an artificial cycle or induced ovulation cycle was used. In the artificial cycle, oral estradiol valerate (Progynova, Delpharm Lille) was applied for endometrial preparation on day 2 or 3 of the second menstrual cycle, after oocyte retrieval. Intramuscular progesterone at a dose of 40 mg/d was applied when the endometrial thickness reached 7 mm. In the induced ovulation cycle, 75 IU/d of human menopausal gonadotrophin was utilized until the largest follicle exceeded 18 mm, 10,000 IU of hCG was injected at midnight to trigger ovulation.

In all FET cycles, one or 2 day 3–6 cryopreserved embryos were thawed and transferred. Luteal-phase support with estradiol valerate and intramuscular progesterone for endometrium preparation continued until 10 weeks after conception.

We identified the implanted embryos and defined clinical pregnancy via ultrasonography at 35 days after FET. All pregnancy and neonatal outcomes were assessed by a call back nurse, including live birth, birth weight, preterm birth, and pregnancy loss: ectopic pregnancy, early abortion (<12 weeks), and late abortion (bewteen 12 and 28 weeks).

### Statistical analysis

Data analyses were conducted using the IBM SPSS Statistics Package (IBM SPSS Statistics 21; IBM, USA). Data are presented as mean ± standard deviation, and comparisons were performed using a 2-sample unpaired *t*-test (normal data distribution) or the Kruskal-Wallis H test (skewed data). Percentages were subjected to the paired chi-squared test. Logistic regression analysis was used to evaluate the factors influencing live birth. A *P*-value < 0.05 was considered statistically significant.

## Results

### Patient characteristics

A total of 398 cycles were included in this study. There were 260 and 138 couples in the N-W and O-O groups, respectively. For the wives, the two groups were similar with regard to mean age, and serum estradiol, follicle-stimulating hormone, and testosterone (Table 1). The BMI of the O-O group was significantly higher than that of the N-W (*P* = 0.000). Compared with the N-W patients, the O-O cohort had significantly lower serum LH, prolactin, and anti-Müllerian hormone (AMH) levels, and took significantly longer to conceive. For the husbands, the two groups were similar at sperm density, sperm motility and abnormal sprem rate.

**Table 1:** Characteristics of the patients at baseline

	Normal	Overweight/obese	*P*
Subjects, n	260	138	–
BMI, kg/m^2^	21.1 ± 1.5	26.8 ± 2.3	0.000
Age, y	28.8 ± 2.7	28.9 ± 3.0	0.787
Time to conceive, y	4.0 ± 2.2	4.8 ± 2.5	0.001
Est2radiol, pmol/L	178.2 ± 149.8	150.5 ± 68.7	0.179
FSH, U/L	4.6 ± 1.2	4.4 ± 1.3	0.102
LH, U/L	7.7 ± 4.7	6.3 ± 4.3	0.002
Testosterone, nmol/L	1.7 ± 0.6	1.9 ± 1.4	0.716
Prolactin, nmol/L	1.9 ± 5.6	1.2 ± 2.8	0.003
AMH, ng/mL	11.1 ± 5.5	9.6 ± 5.7	0.003
Sperm density, × 10^6^ /mL	69.8 ± 54.6	66.4 ± 55.5	0.561
Sperm motility (a + b), %	50.2 ± 19.9	48.0 ± 19.0	0.296
Abnormal sperm rate, %	94.9 ± 7.8	96.3 ± 4.3	0.054

*FSH* follicle-stimulating hormone, *LH* Luteinizing hormone

### Ovarian stimulation, oocyte retrieval, and frozen embryo transfer

Compared with the N-W patients, the O-O group required significantly more days of ovarian stimulation, a higher total dose of gonadotrophin (Gn), and higher Gn/kg (Table 2). However, the applied doses of GnRH-ant did not differ significantly between the two patient groups.

**Table 2:** Outcomes of COH

	Normal	Overweight/obese	*P*
Subjects, n	260	138	–
OHSS, n (%)	7 (2.7)	2 (1.4)	0.725
ICSI, n (%)	17 (6.5)	5 (3.6)	0.258
Ovarian stimulation, d	10.3 ± 2.3	11.2 ± 3.2	0.003
Gn dose, IU	1416.9 ± 533.7	1952.7 ± 747.9	0.000
Gn dose per kg, IU	26.8 ± 9.4	29.2 ± 11.0	0.023
GnRH-ant dose, mg	1.5 ± 0.6	1.5 ± 0.7	0.493
On hCG trigger day			
Follicles, n^a^	2.9 ± 3.9	2.6 ± 3.7	0.528
Estradiol, pmol/L	17,576.9 ± 2005.3	16,804.5 ± 3153.0	0.001
LH, U/L	2.1 ± 4.8	2.8 ± 4.5	0.061
Prolactin, nmol/L	3.9 ± 3.2	3.8 ± 2.3	0.655
Endometrial thickness, mm	9.7 ± 2.0	9.7 ± 2.4	0.418
Before FET			
Estradiol, pmol/L	2365.5 ± 1646.2	1924.7 ± 1484.8	0.010
LH, U/L	9.3 ± 6.9	8.3 ± 8.1	0.222
Prolactin, nmol/L	1.2 ± 2.6	1.1 ± 2.3	0.615
Endometrial thickness, mm	8.8 ± 6.9	8.4 ± 1.6	0.878
Oocytes retrieved, n	23.0 ± 9.8	22.1 ± 9.7	0.562
Good-quality embryos on day 3, n	4.5 ± 3.8	3.9 ± 3.4	0.113
Embryos transferred, n (%)			
Day 3	56 (21.5)	32 (23.4)	0.786
Day 4	7 (2.7)	6 (4.4)	
Day 5	173 (66.5)	87 (63.5)	
Day 6	24 (9.2)	12 (8.8)	
Embryos successfully transferred, n (%)			
One	23 (8.8)	16 (11.6)	0.381
Two	237 (91.2)	122 (88.4)	

*LH* Luteinizing hormone, *OHSS* ovarian hyperstimulation syndrome

^a^Follicles > 18 mm

On the day of hCG trigger, the O-O and N-W groups were similar with regard to the number of follicles > 18 mm, serum luteinizing hormone, prolactin, and endometrial thickness; and also had similar numbers of oocytes retrieved and good-quality embryos on day 3. While the serum estradiol in O-O groups was significant lower than N-W group. The percentages of patients with ovarian hyperstimulation syndrome or underwent ICSI were similar between the 2 groups. At the time of FET, the serum luteinizing hormone, prolactin, endometrial thickness and quantity and grade of transferred frozen-thawed embryos of the 2 groups were similar. While the serum estradiol in O-O groups was significant lower than N-W group.

### Pregnancy outcomes

Compared with the N-W patients, those in the O-O group had significantly lower rates of embryo implantation, live birth, and live birth of twins; and a significantly higher rate of late abortion (Table 3). The following rates of pregnancy-related outcomes of the 2 groups were similar: clinical pregnancy, live births of singletons, ectopic pregnancy, and early abortion. The birth weights of singleton and twins were also similar between the 2 groups.

**Table 3:** Live birth, pregnancy, and pregnancy loss

	Normal	Overweight/obese	*P*
Subjects, n	260	138	–
Embryo implantation, n (%)	290/497 (58.4)	124/260 (47.7)	0.006
Clinical pregnancy, n (%)	186/260 (71.5)	91/138 (65.9)	0.254
Live birth, n (%)	158 /260 (60.8)	66/138 (47.8)	0.015
Singleton	80 (30.8)	51 (37.0)	0.219
Twin	78 (30.0)	15 (10.9)	0.000
Preterm birth, n (%)	57/156 (36.5)	16/66 (24.2)	0.086
Birth weight, kg			
Singleton	3.1 ± 0.6	3.3 ± 0.6	0.167
Twin	2.4 ± 0.5	2.3 ± 0.5	0.383
Pregnancy loss, n (%)			
Ectopic pregnancy	3 (1.6)	1 (1.1)	1.000
Early abortion	18 (9.7)	14 (15.4)	0.167
Late abortion	7 (3.8)	10 (11.0)	0.030

### Logistic regression analysis of independent factor with live birth

We conducted a logistic regression analysis to determine the independent factors associated with live birth (Table 4). We found that the following were not important predictors of clinical outcomes: age; duration of attempt to conceive; number of days embryos were transferred; number of embryos transferred, and endometrial thickness before FET. However, BMI was a risk factor for lower probability of live birth.

**Table 4:** Logistic regression analysis potential factors associated with live birth

	OR (95% CI)	*P*
BMI	0.925 (0.868, 0.986)	0.017
Age	0.946 (0.876, 1.021)	0.154
Duration of attempt to conceive	1.029 (0.939, 1.128)	0.535
Number of days of embryo transferal	0.897 (0.756, 1.065)	0.214
Number of embryos transferred	1.142 (0.582, 2.242)	0.700
Endometrial thickness before FET	1.040 (0.925, 1.169)	0.514

*OR* odds ratio, *CI* confidence interval

## Discussion

This study investigated the efficacy of CHO via the GnRH-ant protocol combined with FET for treatment of infertility in O-O women with PCOS. In a study population of 398 women with PCOS, the outcomes of pregnancy of 138 O-O women were retrospectively evaluated with reference to the outcomes of 260 N-W women. The results indicated that, compared with the N-W patients, the O-O group had significantly lower rates of embryo implantation, live birth, and live birth of twins, and a significantly higher rate of late abortion. The logistic regression analysis indicated that, of the factors analyzed, only BMI was an independent factor affecting the probability of live birth.

PCOS and overweight/obese are common metabolic disorders associated with subfertility [1–6], a recent study observed no significant differences in clinical pregnancy rates between patients with PCOS and those without this syndrome [14]. This appears to indicate that an overweight/obese condition supersedes PCOS as an important factor in female fertility and pregnancy outcomes.

In the year 1998, the World Health Organization determined the overweight and obesity BMI thresholds for Caucasian individuals as 25 kg/m^2^ and 30 kg/m^2^, respectively [15]. In 2002, China’s Obesity Working Group proposed adult BMI thresholds of 24.0 and 28 kg/m^2^ to demarcate an overweight or obese status [11, 12]. In the current study with a Chinese population, we applied a BMI of 24.0 kg/m^2^ as the demarcation for analysis.

Women in N-W group (*n* = 265) are almost two times more than O-O group (*n* = 138) in our study. This result verified a global study showed the percentage of N-W women (63%) are twice as many as O-O women (32%) in China by 2010. While in high-income English-speaking countries and central Afria, the percentages of N-W and O-O are 37% versus 60% and 66% versus 26%, respectively [16]. The overweight/obese is linked to ethnicities, regions, and incomes.

A retrospective cohort study showed that an overweight BMI was no more associated with poor implantation or live-birth rates than normal BMI [17]. However, data regarding the outcomes of IVF/ICSI for patients with PCOS that consider BMI have been inconsistent. For example, O-O patients with PCOS have been reported to have fewer retrieved oocytes, increased rates of miscarriage rates, and lower rates of clinical pregnancy, compared with N-W PCOS patients [18–20].

Serum luteinizing hormone, prolactin, and AMH levels putatively reflect ovarian reserve, whereby decreases correlate with reduced quantity and quality of oocytes [21, 22]. In the present study, the GnRH-ant protocol was used to implement COH. Previous study indicated that there are follicle stimulating hormone receptor (FSHR) and estradiol receptor (ER) localized on adipocyte [23, 24]. As a result, compared with the N-W group, the O-O women required significantly higher doses of Gn, and Gn/kg, and longer periods of ovarian stimulation to retrieve the same number of oocytes and good-quality embryos on day 3. The O-O women with significantly lower serum estradiol both on hCG trigger day and before FET. This result is similar to that of Orvieto et al. [19], in which under the GnRH-ant protocol, PCOS patients with BMI ≤ 25 kg/m^2^ used significantly fewer gonadotrophin ampoules to achieve similar periods of ovarian stimulation and numbers of oocytes retrieved.

In the current study, before FET endometrial preparation was performed under conditions of natural, artificial, or induced ovulation cycles. As Yuan et al. [25] discovered, endometrial thickness is an important and independent predictor of pregnancy outcomes after IVF/ICSI treatment. A meta-analysis also showed that an endometrial thickness cut-off of 7 mm (frequently used) correlates with lower chances of pregnancy [26]. In our data, the endometrial thickness before FET reached 8.4 mm, and was not significantly different between the 2 groups. This reduced the possibility that endometrial factors might have disturbed embryo implantation.

Other factors that influence pregnancy outcomes are quantity and quality of transferred embryos. A meta-analysis that compared blastocyst transfer with cleavage-stage embryo transfer showed that neither had an advantage with regard to rates of clinical pregnancy, ongoing pregnancy, or live births [27]. In our current study, we transferred similar numbers of cleavage-stage or blastocyst frozen-thawed embryos between the 2 cohorts.

Although we found that the O-O patients had significantly lower rates of embryo implantation, live births, live birth twins, compared with the N-W patients, another Chinese scholar found that women with PCOS and BMI ≥ 24 kg/m^2^ had lower clinical pregnancy rates but similar miscarriage and live birth rates [28]. That study used the standard long GnRH agonist protocol for COH with fresh embryo transfer. In the fresh transfer cycle, women were placed under excessive estrogen and prolactin, which results in ovarian stimulation and can also affect the embryo and endometrial receptivity [29]. In contrast, we chose the GnRH-ant protocol and transfer of frozen-thawed embryos. This is more suitable for patients with PCOS [7, 8], especially because the uterine environment is more natural in the frozen replacement cycle.

In our study, the rate of late abortion was higher in the O-O patients compared with the N-W. We consider that the O-O condition affects embryo implantation.

Our study has the following limitations. First, this is a retrospective study and our results need to be confirmed by randomized controlled trials. Second, in this study we only measured endometrial thickness to evaluate its receptivity. However, to ensure best endometrial receptivity, additional indices such as endometrial patterns and endometrial blood flow should be included. The collection of detailed information regarding pregnancy and neonatal complications will allow us to analyze better the effects of being overweight or obese.

## Conclusions

In conclusion, although for this population of PCOS patients we applied COH via the GnRH-ant protocol and transferred frozen-thawed embryos, the condition of being overweight or obese remained a risk factor of reduced rates of embryo implantation, live birth, and live birth of twins, and increased the risk of late abortion. These results show that weight loss is particularly important for patients with PCOS. Further studies are needed with larger sample sizes, and thorough follow-ups to assess the long-term safety for neonates.
